# Comprehensive Analysis of 5-Methylcytosine (m^5^C) Regulators and the Immune Microenvironment in Pancreatic Adenocarcinoma to Aid Immunotherapy

**DOI:** 10.3389/fonc.2022.851766

**Published:** 2022-03-31

**Authors:** Ronglin Wang, Yongdong Guo, Peixiang Ma, Yang Song, Jie Min, Ting Zhao, Lei Hua, Chao Zhang, Cheng Yang, Jingjie Shi, Liaoliao Zhu, Dongxue Gan, Shanshan Li, Junqiang Li, Haichuan Su

**Affiliations:** Department of Oncology, Tangdu Hospital, Air Force Medical University, Xi'an, China

**Keywords:** pancreatic adenocarcinoma, m^5^C regulators, RNA modification, immune microenvironment, immunotherapy

## Abstract

**Background:**

Pancreatic adenocarcinoma (PAAD) is one of the most malignant cancers and has a poor prognosis. As a critical RNA modification, 5-methylcytosine (m^5^C) has been reported to regulate tumor progression, including PAAD progression. However, a comprehensive analysis of m^5^C regulators in PAAD is lacking.

**Methods:**

In the present study, PAAD datasets were obtained from the Gene Expression Omnibus (GEO), The Cancer Genome Atlas (TCGA), International Cancer Genome Consortium (ICGC), and ArrayExpress databases. The expression pattern of m^5^C regulators were analyzed and patients were divided into different m^5^C clusters according to consensus clustering based on m^5^C regulators. Additionally, m^5^C differentially expressed genes (DEGs) were determined using Limma package. Based on m^5^C DEGs, patients were divided into m^5^C gene clusters. Moreover, m^5^C gene signatures were derived from m^5^C DEGs and a quantitative indicator, the m^5^C score, was developed from the m^5^C gene signatures.

**Results:**

Our study showed that m^5^C regulators were differentially expressed in patients with PAAD. The m^5^C clusters and gene clusters based on m^5^C regulators and m^5^C DEGs were related to immune cell infiltration, immune-related genes and patient survival status, indicating that m^5^C modification play a central role in regulating PAAD development partly by modulating immune microenvironment. Additionally, a quantitative indicator, the m^5^C score, was also developed and was related to a series of immune-related indicators. Moreover, the m^5^C score precisely predicted the immunotherapy response and prognosis of patients with PAAD.

**Conclusion:**

In summary, we confirmed that m^5^C regulators regulate PAAD development by modulating the immune microenvironment. In addition, a quantitative indicator, the m^5^C score, was developed to predict immunotherapy response and prognosis and assisted in identifying PAAD patients suitable for tailored immunotherapy strategies.

## Introduction

Pancreatic adenocarcinoma (PAAD) is one of the most malignant cancers worldwide. Because of the lack of an early diagnosis strategy, distant tumor metastasis often occurs, after which patients are not eligible for surgical resection. The five-year survival rate of patients with PAAD is approximately 5% (1). Chemotherapy and radiotherapy are the standard treatments for patients with PAAD and improve prognosis to some extent. Immunotherapy is a promising treatment to prolong the prognosis of PAAD and improve the quality of life of PAAD patients, although unfortunately, a large number of PAAD patients are resistant to immunotherapy (2). Uncovering the underlying mechanisms and validating novel response indicators can assist in the clinical application of immunotherapy.

Many factors have been reported to modulate immunotherapy sensitivity, and the tumor microenvironment (TME) is one of the most critical factors (3). The TME is composed of immune cells, surrounding fibroblasts, intercellular stroma, microvessels, and infiltrating biological molecules. Among these, infiltrating immune cells play a central role in modulating the immunotherapy response. It has been reported that an increased number of cytotoxic T cells and dendritic cells and a decreased number of Treg cells help limit tumor growth (4–6). Critical factors or pathways regulating immune cell infiltration are related to immunotherapy response and survival status. For example, m^6^A (N6-methyladenosine) modification was reported to modulate the TME, and in models, m^6^A regulators were perfect indicators for predicting immunotherapy response in previous and our studies (7–9). More indicators urgently need to be discovered and validated.

5-Methylcytosine (m^5^C) is a prevalent RNA modification. Similar to m^6^A modification, m^5^C modification has three types of regulators: methyltransferases (writers), demethylases (erasers), and binding proteins (readers). By recognizing specific motifs, regulators control the m^5^C modification level. m^5^C modification has been reported to modulate cancer development by regulating target RNA stability, translocation, and translation (10). In bladder cancer, the m^5^C modification writer NSUN2 was shown to promote cancer development by regulating HDGF expression in an m^5^C modification-dependent manner (11). In lung adenocarcinoma, the long noncoding RNA (lncRNA) LINC00312 targets YBX1 to promote cancer migration and vasculogenic mimicry (12).

m^5^C modification is also related to the TME. In CD4^+^ T cells, dysregulated m^5^C modification was examined, and target mRNAs were related to systemic lupus erythematosus (SLE) pathogenesis (13). In triple-negative breast cancer, m^5^C modification regulators were found to affect the TME and predict patient prognosis (13). In lung adenocarcinoma, 14 lncRNAs were found to be regulated by m^5^C modifications. Moreover, these lncRNAs were found to modulate the TME and predict the survival status of patients (14). Additionally, some bioinformatics analyses also indicated m^5^C modification regulates the TME and affects tumor progression (15–19). In PAAD, the decreased level of the m^5^C regulator NSUN6 represses tumor proliferation (20). However, to date, there has been no comprehensive analysis of m^5^C modifications in PAAD. The role of m^5^C regulators in the PAAD TME and PAAD development is largely unknown.

**Figure 1:**
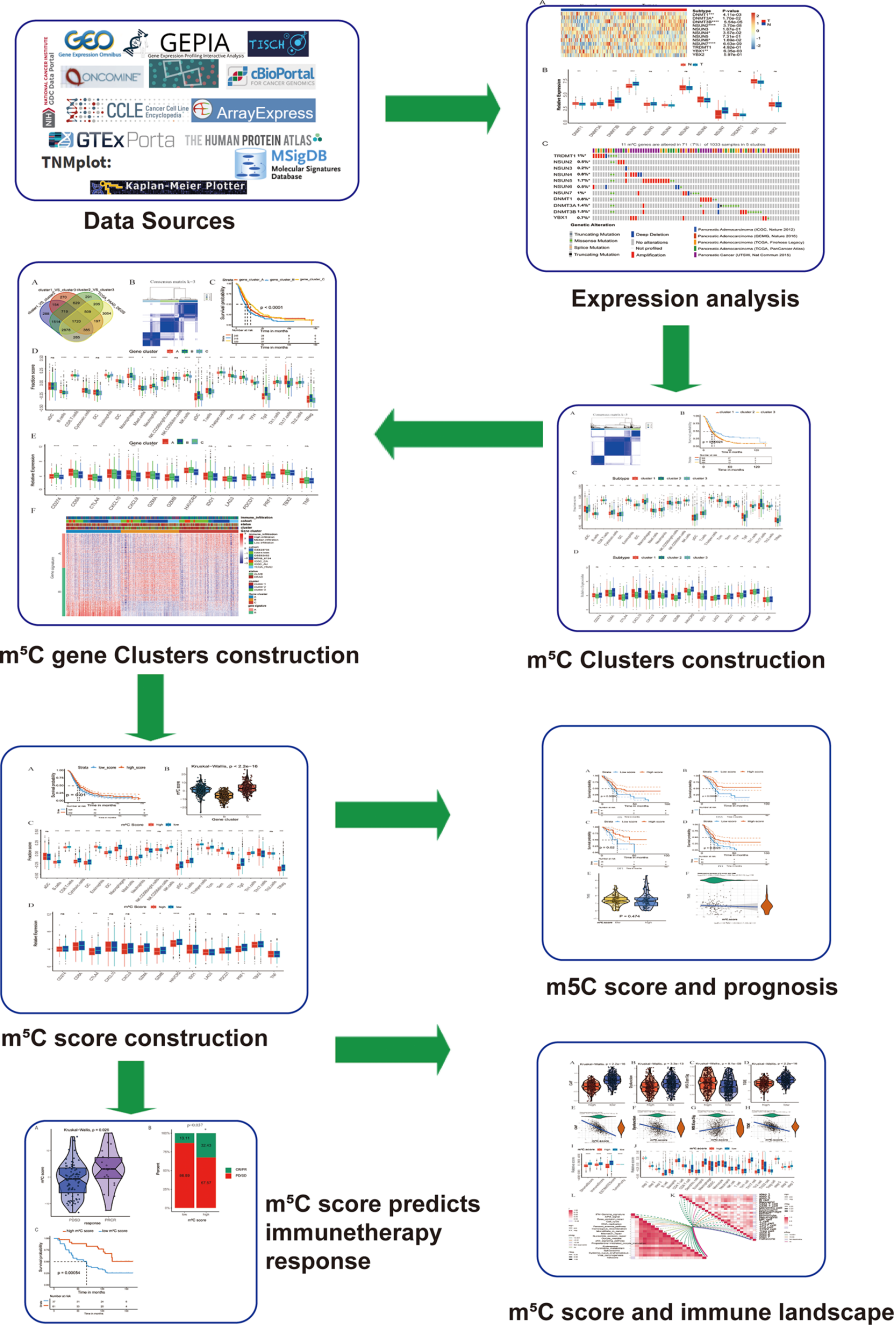
Workflow of the present study. To explore the function and underlying mechanism of m^5^C regulators in PAAD immunotherapy response and development, we downloaded and integrated multiple PAAD datasets. The expression pattern and genetic alteration of m^5^C regulators were analyzed firstly. Then patients were divided into m^5^C clusters and gene clusters using unsupervised clustering method. Bioinformatic analysis of m^5^C clusters and gene clusters indicated they were related with immune microenvironment of PAAD. Additionally, a quantitative indicator m^5^C score was constructed, which reflected immune microenvironment, predicted immunotherapy response and correlated with patients’ prognosis.

In the present study, we found that m^5^C regulators were differentially expressed between PAAD tissues and corresponding normal tissues. Stratification analyses based on m^5^C regulators and the m^5^C differentially expressed genes (DEGs) between m^5^C clusters revealed that m^5^C modification was related to the TME and associated with the survival status of PAAD patients. Additionally, the m^5^C score was determined based on the m^5^C DEGs for PAAD patients to characterize the PAAD TME. The m^5^C score precisely predicted the immunotherapy response and prognosis of patients with PAAD. The workflow of the present study is shown in **Figure 1**.

## Materials and Methods

### Collection and Preprocessing of PAAD Datasets

The integrated dataset containing seven independent PAAD datasets has been described in our previous studies (9). We collected the PAAD datasets from the Gene Expression Omnibus (GEO) (https://www.ncbi.nlm.nih.gov/geo/), The Cancer Genome Atlas (TCGA) (https://www.cancer.gov/about-nci/organization/ccg/research/structural-genomics/tcga), International Cancer Genome Consortium (ICGC) (https://dcc.icgc.org/), and ArrayExpress databases (https://www.ebi.ac.uk/arrayexpress). Patients without survival information were eliminated from the integrated datasets. Seven independent datasets, namely, the GSE28735, GSE57495, GSE62452, MTAB-6134, TCGA-PAAD, ICGC-AU and ICGC-CA datasets, were collected as the training cohort, and the datasets included 930 patients with PAAD. The details of these seven datasets were summarized in sheet 1, 3, 8 and 9 of **Supplementary Table 1**. XLX (In the supplementary data). For it’s difficult to find more PAAD datasets with clinical data, we validated our results in three datasets of the seven datasets. The ICGC-AU and ICGC-CA datasets, including 295 patients, and the TCGA-PAAD dataset were used as the validation cohort for further analysis. The ComBat method from the “SVA” package was used to correct the batch effects from nonbiological technical biases (21). In this process, some information was lost, and we could not detect the expression of YBX2 in the integrated dataset with 930 PAAD patients.

### Examination of m^5^C Regulator Expression

Twelve m^5^C regulators were determined based on previous studies (14, 22). For GSE62165 dataset has no clinical data, it’s excluded from the integrated dataset. For GSE62165 has both PAAD and corresponding normal tissues, it was used to analyze the mRNA expression of these twelve regulators. Additionally, the protein expression of these regulators was acquired from the Human Protein Atlas (HPA) database (http://www.proteinatlas.org/). Genetic alteration was thought to modulate gene expression. To uncover the underlying mechanism of m^5^C regulators expression pattern, the cBioPortal database (http://www.cbioportal.org/) was used to examine m^5^C regulator genetic alterations, including amplification, deep deletion, and missense mutations.

### Consensus Clustering Based on m^5^C Regulators

Based on m^5^C regulator expression levels, the patients with PAAD were divided into different clusters using the unsupervised clustering package “ConsensusClusterPlus” (23). To validate the classification stability, this process was repeated 1000 times. The principal component analysis was performed using the “PCA” package in R software.

### Analysis of Immune Cell Infiltration by Single-Sample Gene Set Enrichment Analysis (ssGSEA)

The ssGSEA algorithm was used to analyze the infiltration level of 24 immune cell types that play critical roles in cancer immunity (24). The marker genes for each type of immune cell were determined according to previous studies (25–27). Based on the immune infiltration level, the patients in the dataset were divided into high-, moderate- and low-infiltration groups using unsupervised clustering.

### Detection of ESTIMATE and the Tumor Immune Dysfunction and Exclusion (TIDE) Scores

The ESTIMATE algorithm was used to calculate the immunoscore and stromal score (28). By analyzing specific mRNA expression, we also determined tumor cellularity and tumor purity (28). The TIDE score was determined using the TIDE algorithm (29). A high TIDE score indicates that cancer cells are more likely to resist immunotherapy and promote immune evasion.

### Extraction of m^5^C Relative Differentially Expressed Genes (m^5^C DEGs) and Calculation of m^5^C Score

Firstly, we extracted differently expressed genes (DEGs) between normal and tumor tissues in PAAD patients from GEPIA database (http://gepia2.cancer-pku.cn/#index) (30). Then, we obtained 2439 differentially expressed genes among three m^5^C clusters by using limma packages in R software and an adjusted p<0.05 was set as the cutoff value (31). Lastly, we took intersection from DEGs (from GEPIA) and 2439 DEGs (from m^5^C clusters) to increase the specificity of the target genes. Generally, we obtained 1720 merged differentially expressed m^5^C-related genes (m^5^C DEGs). We performed unsupervised clustering of these m^5^C DEGs in training cohort and divided the cohort into three clusters, named gene cluster A, B, and C. The gene signatures that were positively correlated with gene cluster were named as m^5^C gene signature A, and the rest m^5^C DEGs were termed as m^5^C gene signature B. We performed functional enrichment analysis of m^5^C gene signature A and B using over representation analysis. In order to reduce the noise or redundant genes, we used the Boruta algorithm to perform dimension reduction in the m^5^C gene signature A and B (32).

To construct the m^5^C score, principal component analysis (PCA) was utilized to construct m^5^C score from the m^5^C gene signature A and B. (1) the PC1A represent the first component of m^5^C gene signature A, and (2) the PC1B represent the first component of m^5^C gene signature B. m^5^C score = ∑PC1A-∑PC1B. 930 patients in training cohort were stratified into two subgroups as high- and low- m^5^C score by using surv_cutpoint function in the survminer package in R software.

### Establishment of the Immunotherapy Cohort

GSE91061 was downloaded to explore the ability of the m^5^C score to predict immunotherapy response. The GSE91061 dataset contains patients with melanoma receiving PD-1 treatment (33).

### Analysis of Functional Mechanisms

Gene set variation analysis (GSVA) was used to explore the underlying characteristic of the high and low m^5^C score groups of patients with PAAD (34). The hallmark gene set (h.all.v6.2.entrez.gmt) was obtained from the Molecular Signatures Database (MSigDB). The cancer immunity cycle and immunotherapy-related pathways were detected according to previous studies (35, 36). Additionally, we obtained cancer driver genes from the Integrative OncoGenomics database (IntOGen, https://www.intogen.org/) (37).

### Examination of Chemotherapy Response

The "pRRophetic" package in R was used to analyze the chemotherapy response (IC50) based on the Genomics of Drug Sensitivity in Cancer (GDSC) database (38). IC50 indicates the effects of the tested drugs on suppressing cell growth. If p value < 0.05, it indicates the sensitivity of the drug is different between high m^5^C and low m^5^C groups

### Western Blot

PAAD tissues and control tissues were obtained from patients who underwent surgical resection at Tangdu Hospital. Written informed consent was obtained from every patient, and all procedures performed in patients were approved by the Research Ethics Committee of Tangdu Hospital. The tissues were cut into small pieces using scissors and lysed with lysis buffer (C1053-100, Applygen, Beijing, China) supplemented with protease and phosphatase inhibitors (04906837001 and 04693159001, Roche, Basel, Switzerland). After incubation on ice for 0.5 h, the samples were centrifuged, and the supernatant was collected. The protein concentration was analyzed using a BCA kit (23225, Thermo, MA, US). The samples were analyzed using 10% SDS gel electrophoresis and transferred to polyvinylidene fluoride (PVDF) membranes. The blots were incubated with primary antibodies at 4°C overnight. As YBX2 was not detectable in the integrated dataset, we only examined 11 m5C modification regulators in PAAD and corresponding tissues. Anti-DNMT1 (A19679), anti-DNMT3A (A19659), anti-DNMT3B (A11079), anti-NSUN2 (A3443), anti-NSUN3 (A12892), anti-NSUN4 (A14983), anti-NSUN5 (A5992), anti-NSUN6 (A7205), anti-TRDMT1 (A10535), anti-YBX1 (A7704) and anti-GAPDH (A19056) antibodies were purchased from Abclonal (Wuhan, China). The anti-NSUN7 (17546-1-AP) antibody and secondary HRP antibodies (SA00001-1 and SA00001-2) were purchased from Proteintech (Wuhan, China). The blots were washed with TBST buffer three times and incubated with the corresponding secondary HRP antibodies for 2 h at room temperature. Finally, the blots were assessed using a GelDoc XR+ gel imager (Bio-Rad, USA).

### Statistical Analysis

R-4.0.4 was used to perform the statistical analyses in this study. For quantitative data, Student’s t tests were used for normally distributed variables, and the Wilcoxon rank-sum test was used for nonnormally distributed variables. To compare more than two groups, Kruskal–Wallis tests and one-way ANOVA were used as nonparametric and parametric methods, respectively. The Kaplan–Meier method and the Cox proportional hazards model were used to analyze survival. The “survival” and “Survminer” packages were used to examine the association between the factors and prognosis. The surv-cutpoint function from the “survival” package was applied to divide the included PAAD samples into different subtypes. Correlation analysis in the present study was performed with Pearson or Spearman correlation coefficients and using the “corrplot” package in R software. All comparisons were two-sided with an alpha level of 0.05, and the Bonferroni method was used to control the false discovery rate (FDR) for multiple hypothesis testing.

## Results

### Landscape of Expression and Genetic Alterations of m^5^C Regulators in PAAD

**Figure 2:**
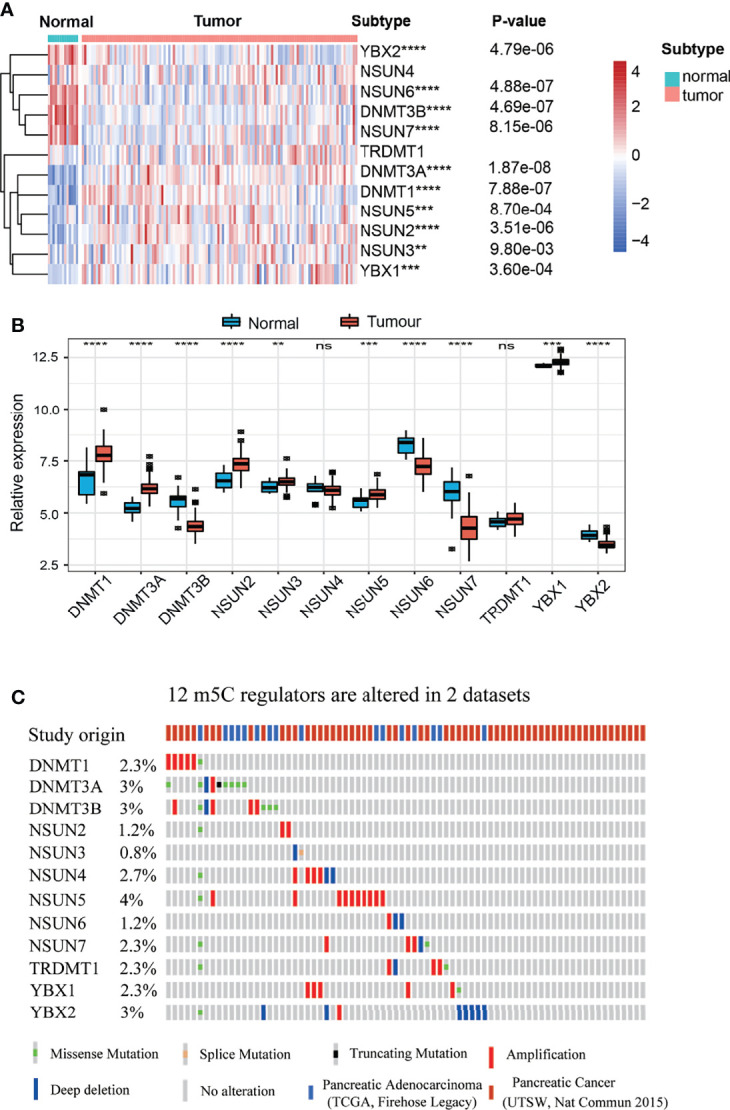
Landscape of expression and genetic alterations of m^5^C regulators in PAAD. **(A, B)** The mRNA expression levels of 12 m^5^C regulators between normal and PAAD samples in the GSE61625 dataset. **(C)** Fifty-one of the 259 PAAD patients in 2 studies had genetic alterations of 12 m^5^C regulators in the cBioPortal database. ** represents p value < 0.01, *** represents p value < 0.001, **** represents p value < 0.0001, ns means "not statistically significant".

m^5^C regulators play a critical role in maintaining target RNA stability, translation, or translocation (39). However, the landscape of expression and genetic alterations of m^5^C regulators has not been elucidated in PAAD. We analyzed the expression pattern of m^5^C regulators in GSE62165, which contains 131 PAAD tissues and adjacent normal tissues. The present results indicated that the levels of DNMT3B, NSUN6, NSUN7 and YBX2 mRNA were significantly decreased while the levels of DNMT1, DNMT3A, NSUN2, NSUN3, NSUN5 and YBX1 mRNA were significantly increased in PAAD patients (**Figures 2A, B**). We also analyzed the protein expression of m^5^C regulators using the Human Protein Atlas (HPA) database (**Supplementary Figure 1**). Our results showed that NSUN6 levels were decreased in PAAD, in line with previously established expression patterns (20). Genetic alterations are critical factors that influence disease progression (40). We examined the genetic alterations of these regulators in cBioPortal database. These results indicated that 51 of the 259 (19.69%) PAAD patients in 2 studies experienced genetic alterations of 12 m^5^C regulators (**Figure 2C**). The results confirmed that m5C regulators are differentially expressed in PAAD patients, which may play a critical role in PAAD progression.

### Identification of Consensus Clusters and Analysis of Correlations Between the Clusters and the Immune Microenvironment

**Figure 3:**
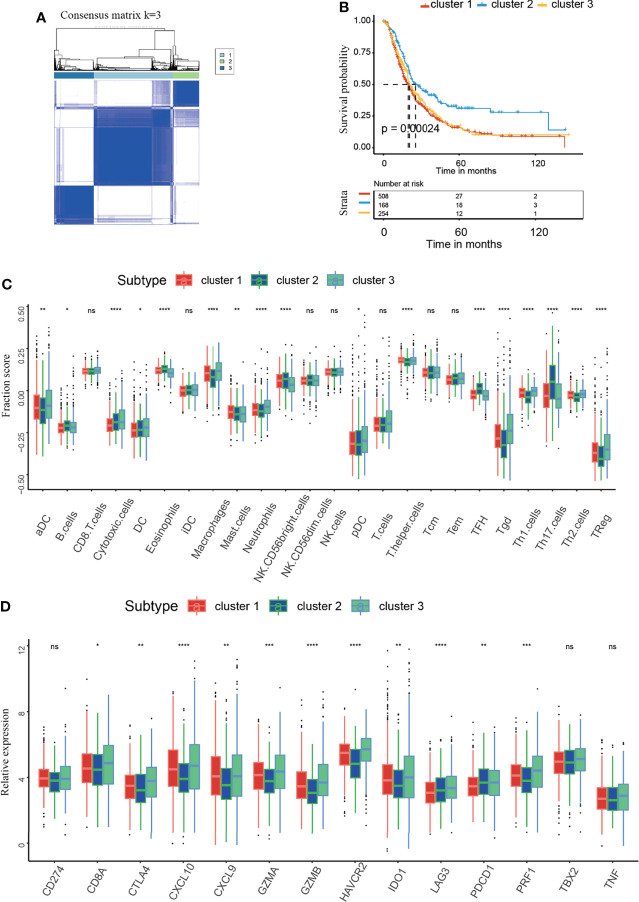
Identification of the consensus clusters and analysis of the correlation between clusters and the immune microenvironment. **(A)** Consensus clustering matrix for k=3. **(B)** Kaplan–Meier curves of overall survival (OS) in m^5^C clusters 1-3 of patients with PAAD. **(C)** Immune cell infiltration in m^5^C clusters 1-3. **(D)** Expression levels of immune activation-related genes and immune checkpoint-related genes in m^5^C clusters 1-3. * represents p value < 0.05, ** represents p value < 0.01, *** represents p value < 0.001, **** represents p value < 0.0001, ns means "not statistically significant".

As shown in our previous studies, we conducted further analysis in the integrated dataset with 930 PAAD patients, which was composed of seven dependent PAAD dataset with clinical data (9). According to the similarity displayed by the m^5^C regulator expression levels, k = 3 was identified, with optimal clustering stability from k = 2 to 9 (**Supplementary Figure 2**). The integrated dataset was divided into three independent m^5^C clusters (**Figure 3A**). As shown in **Figure 3B**, m^5^C clusters were significantly related to PAAD patient survival status. m^5^C cluster 2 had the best overall survival (OS) when compared to clusters 1 and 3 (**Figure 3B**). This result confirms that m^5^C regulators are critical factors influencing the prognosis of PAAD. It has been reported m^5^C modification is involved in immune microenvironment regulation (22). Then we analyzed the correlation between m^5^C regulators and immune microenvironment of PAAD. The result indicated immune cell infiltration was differentially distributed between the m^5^C clusters. Specifically, m^5^C cluster 2 had the highest proportions of B cells, NK CD56 bright cells, Tfh cells, Th17 cells and the lowest proportion of Treg cells when compared to clusters 1 and 3 (**Figure 3C**). These cells have been reported to activate the immune response and kill cancer cells (41). Additionally, we analyzed the relationships between the m^5^C clusters and immune-related genes. In the present study, *CD274*, *CTLA4*, *HAVCR2*, *IDO1*, *LAG3*, and *PDCD1* were included in the immune checkpoint-related signature, and *CD8A*, *CXCL10*, *CXCL9*, *GZMA*, *GZMB*, *PRF1*, *TBX2*, and *TNF* were included in the immune activity-related signature (42). The results showed that cluster 2 had the lowest levels of CTLA4 and IDO1, which play an inhibitory role in immune cell activation (**Figure 3D**). These results showed that m^5^C cluster 2 had the most potent immune response when compared with cluster 1 and 3, which partly account for the best prognosis of patients in the m^5^C cluster2. These results confirmed that m^5^C regulators are related to the PAAD immune microenvironment. The tumor immune microenvironment plays a role in the effect of m5C regulators on patient prognosis.

### Identification of m^5^C-Related Gene Clusters and Analysis of the Correlations Between the Gene Clusters and the Immune Microenvironment

**Figure 4:**
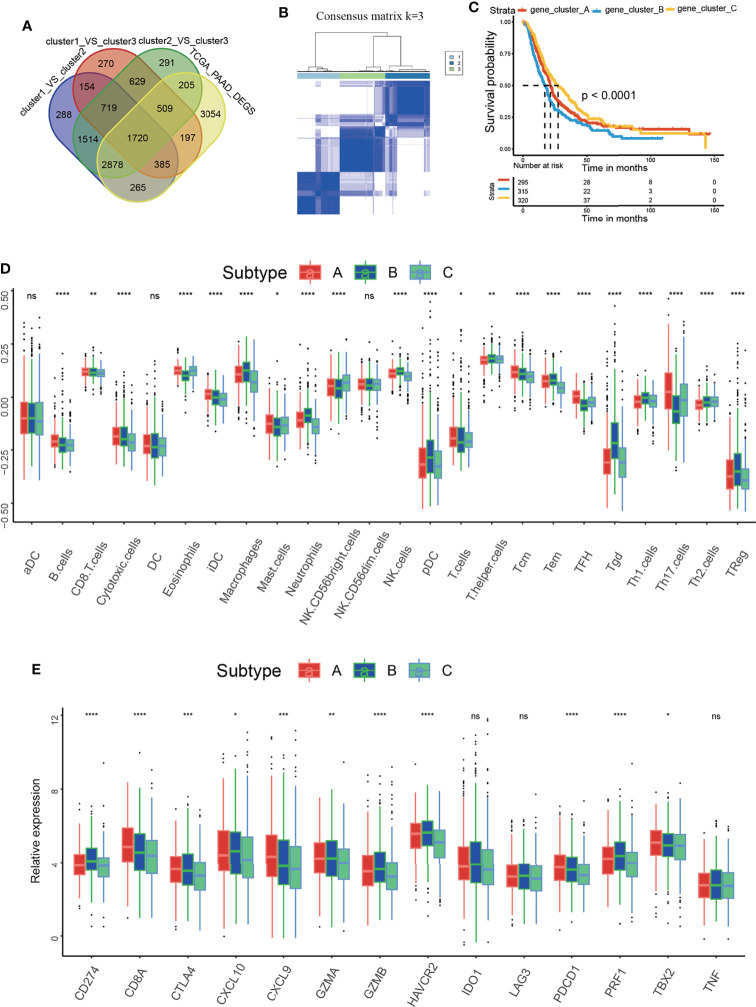
Identification of the m5C-related gene clusters and analysis of the correlation between the gene clusters and the immune microenvironment. **(A)** Venn diagram delineating the DEGs between the three m^5^C clusters and the DEGs between non-tumor and tumor samples from PAAD patients of GEPIA dataset. **(B)** Consensus clustering matrix for k=3. **(C)** Kaplan–Meier curves of overall survival (OS) for patients with PAAD in m^5^C gene clusters A, B and C. **(D)** Immune cell infiltration in m^5^C gene clusters A, B and C. **(E)** Expression of immune activation-related genes and immune checkpoint-related genes in m^5^C gene clusters A, B and C. * represents p value < 0.05, ** represents p value < 0.01, *** represents p value < 0.001, **** represents p value < 0.0001, ns means "not statistically significant".

To further probe into the biological behaviors accounting for the important role of m^5^C regulators in the PAAD TME and PAAD development, we tried to identified the m^5^C DEGs. As described in the method section, DEGs from GEPIA dataset was intersected with DEGs from three m^5^C clusters. Finally, we identified a total of 1720 m^5^C DEGs (**Figure 4A**). Based on the unsupervised clustering of these m^5^C DEGs, 930 PAAD patients were divided into three m^5^C-related gene clusters, namely, m^5^C gene clusters A, B, and C (**Figure 4B** and **Supplementary Figure 3**). The results showed that the m^5^C gene clusters were strongly correlated with survival status: m^5^C gene cluster C had the best OS when compared to clusters A and B (**Figure 4C**). We also analyzed the correlations with immune cell infiltration. m^5^C gene cluster C had the highest proportion of NK CD56bright cells and the lowest proportion of Treg cells (**Figure 4D**). Additionally, these two kinds of immune cells were overlapped with that in m^5^C cluster2. Functionally, NK CD56bright cells are mainly responsible for innate immune response and Treg cells are one of the most canonical immune-suppressive cells in the body (43, 44). Moreover, m^5^C gene cluster C had the lowest levels of CD274, CTLA4 and PDCD1 (**Figure 4E**). These results confirmed m^5^C gene cluster C had the most potent immune response when compared with cluster A and B. Collectively, these results further confirmed that m^5^C regulators are critical factors influencing the PAAD TME and PAAD development. The coherence between the prognostic and TME features in the three gene clusters indicated that this classification was reliable and reasonable.

### Construction of the m^5^C Score and Its Relationship With the Immune Microenvironment

**Figure 5:**
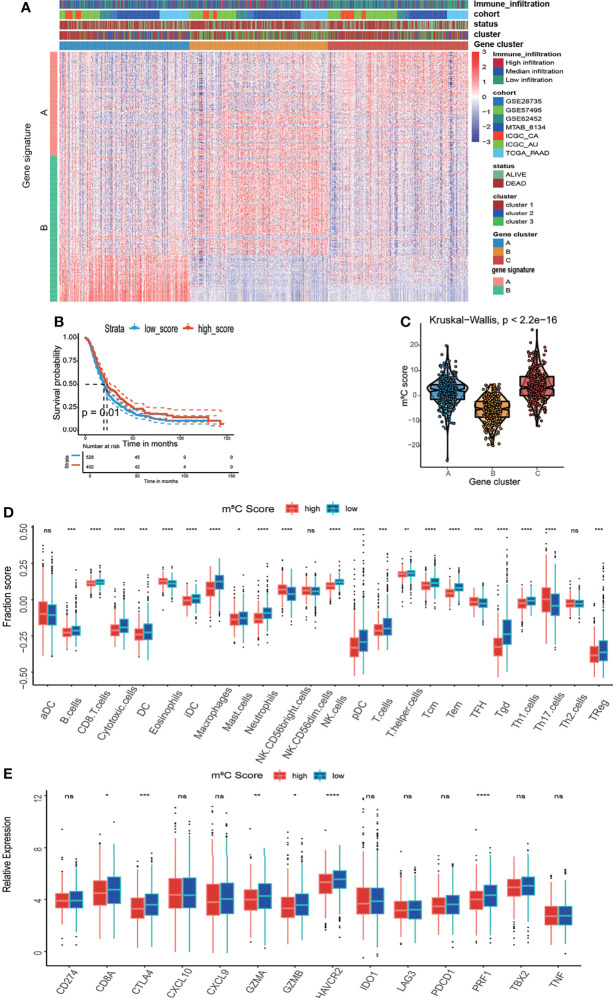
Development of the m^5^C score and assessment of its relationship with the immune microenvironment. **(A)** Unsupervised clustering of 1720 DEGs between the three m^5^C gene clusters and identification of m^5^C gene signature A and B. **(B)** Kaplan–Meier curves of overall survival (OS) for patients with PAAD in the high and low m^5^C score subtypes. **(C)** Correlation between the m^5^C score and the m^5^C gene clusters. **(D)** Immune cell infiltration in the high and low m^5^C score subtypes. **(E)** Expression levels of immune activation-related genes and immune checkpoint-related genes in the high and low m^5^C score subtypes. * represents p value < 0.05, ** represents p value < 0.01, *** represents p value < 0.001, **** represents p value < 0.0001, ns means "not statistically significant".

**Figure 6:**
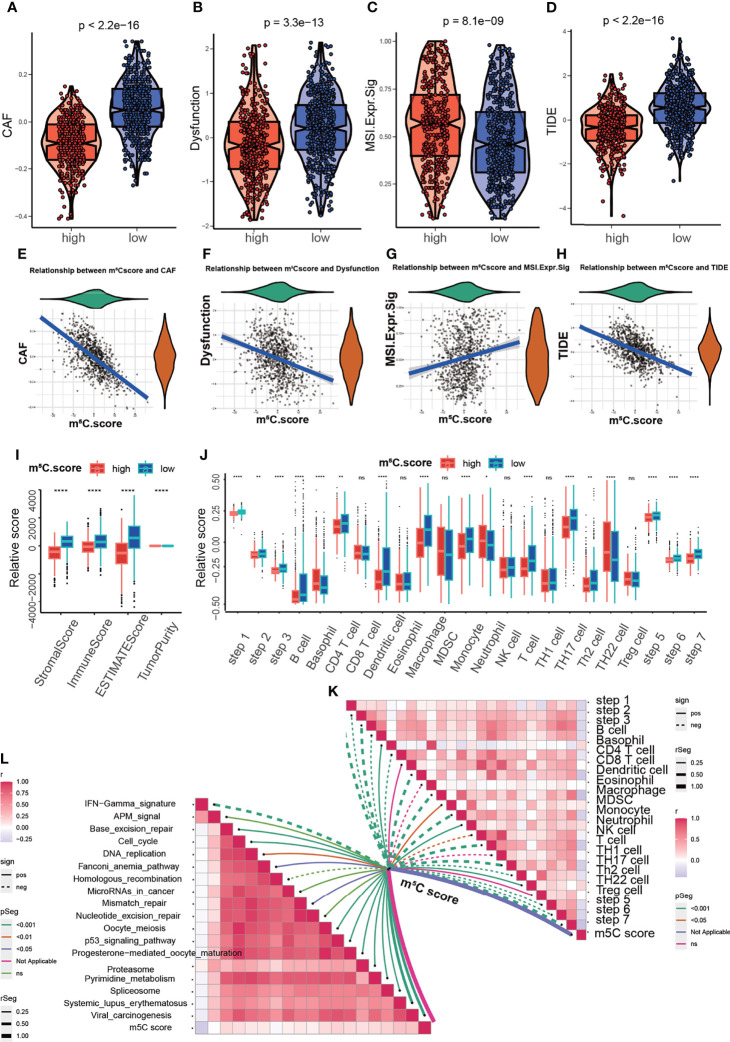
Analysis of the correlation between the m^5^C score and the immune microenvironment. **(A, E)** Correlation between the m^5^C score and cancer-associated fibroblasts (CAFs). CAF t_Student_(928)=-24.87, p=4.69e-105, r_pearson_=-0.63, CI_95%_=[-0.67, -0.59], n_pairs_=930. **(B, F)** Correlation between the m^5^C score and T cell dysfunction. Dysfunction t_Student_(928)=-9.50, p=1.78e-20, r_pearson_=-0.30, CI_95%_=[-0.36, -0.24], n_pairs_=930. **(C, G)** Correlation between the m^5^C score and microsatellite instability (MSI). MSI.Expr.Sig t_Student_(928)=-5.36, p=1.03e-07, r_pearson_=0.17, CI_95%_=[0.11, 0.24], n_pairs_=930. **(D, H)** Correlation between the m^5^C score and the TIDE score. TIDE t_Student_(928)=-16.37, p=3.92e-53, r_pearson_=-0.47, CI_95%_=[-0.52, -0.42], n_pairs_=930. **(I)** Correlation between the m^5^C score and the stromal score, immune score, ESTIMATE score and tumor purity. **(J, K)** Correlation between MBS and the steps of the cancer immunity cycle. **(L)** Correlation between MBS and the enrichment score of immunotherapy-related pathways. * represents p value < 0.05, ** represents p value < 0.01, **** represents p value < 0.0001, ns means "not statistically significant".

To acquire quantitative indicators of m^5^C regulators, a heatmap was generated to visualize the correlation among the m^5^C clusters, m^5^C gene clusters, patient status, and immune infiltration. As shown in the left axis of the heatmap, m^5^C gene signatures that were positively correlated with m^5^C gene cluster were named as m^5^C gene signature A, and the rest m^5^C DEGs were termed as m^5^C gene signature B (**Figure 5A**). This method has been verified in previous study (42). Then the dimension reduction was presented by the Boruta algorithm based on the m^5^C gene signatures A and B and principal component analysis (PCA) was utilized to extract the principal component from the m5C gene signature A and B. (1) the PC1A represent the first component of m5C gene signature A, and (2) the PC1B represent the first component of m5C gene signature B. m5C score = ∑PC1A-∑PC1B. 930 patients in training cohort were stratified into two subgroups as high- and low- m^5^C score by using surv_cutpoint function in the survminer package in R software. The results indicated that higher m^5^C score was related to longer OS than lower score (**Figure 5B**). Consistently, cluster C had the highest m^5^C score and the best OS rate (**Figure 5C**). These results suggested m^5^C score was a potential indicator to predict PAAD prognosis. It has been validated m^5^C modification was related to immune microenvironment of PAAD, we also analyzed the correlation between the m^5^C score, infiltrating immune cells, and immune-related genes. The high m^5^C score group had a higher proportion of NK CD56 bright cells and a lower proportion of Treg cells, which was consistent with the result of m^5^C cluster 2 and gene cluster C (**Figure 5D**). Moreover, the high m^5^C score group had lower levels of CD274, CTLA4, IDO1, LAG3, and PDCD1 expression despite the fact that the differences in some factors were not statistically significant (**Figure 5E**). Additionally, we determined the relationship between the m^5^C score and cancer-associated fibroblasts (CAFs), T cell dysfunction, microsatellite instability (MSI), and the TIDE score. It has been validated an increased number of CAFs promotes cancer development by hindering immune cell infiltration into cancer tissues, while MSI and the TIDE score are precise predictors of chemotherapy and immunotherapy response (45–47). High m^5^C score was found to be related to lower CAF levels, T cell dysfunction, and lower TIDE scores but were also related to higher MSI (**Figures 6A–H**). Moreover, the high m^5^C score group had a lower stromal score, immune score, and ESTIMATE score but higher tumor purity (**Figure 6I**). These results further confirmed m^5^C score was related with immune microenvironment of PAAD. High m^5^C score indicated a better immune response.

The cancer immunity cycle is useful for determining which steps are involved in the immune response (48). Next we aimed to explore the immune steps regulated by the m^5^C score *via* using the cancer immunity cycle tool. The results suggested that the high m^5^C score group had high proportions of basophils and neutrophils (**Figure 6J**). Moreover, we aim to explore the mechanism underlying m^5^C score. The correlation of m^5^C score and immunotherapy-related pathways was analyzed. The m^5^C score was related to base-excision repair, the cell cycle, DNA replication, the p53 signaling pathway and other pathways (**Figures 6K, L**). These results assisted to elucidate the mechanisms of m^5^C score in regulating immune response. To comprehensively validate the utility of the m^5^C score, a heatmap was constructed to delineate the correlation between the m^5^C score and the TME. The m^5^C score was related to the proportions of CD8^+^ T cells, NK cells and B cells, the expression of immune checkpoints and MHC-II, the EMT process, the cell cycle and mismatch repair (**Supplementary Figure 4**). m^5^C modification were closely related with immune microenvironment and participated in immune response modulation *via* multiple signaling pathways.

### Performance of the m^5^C Score in Predicting Immunotherapy Response and Chemotherapy Response

**Figure 7:**
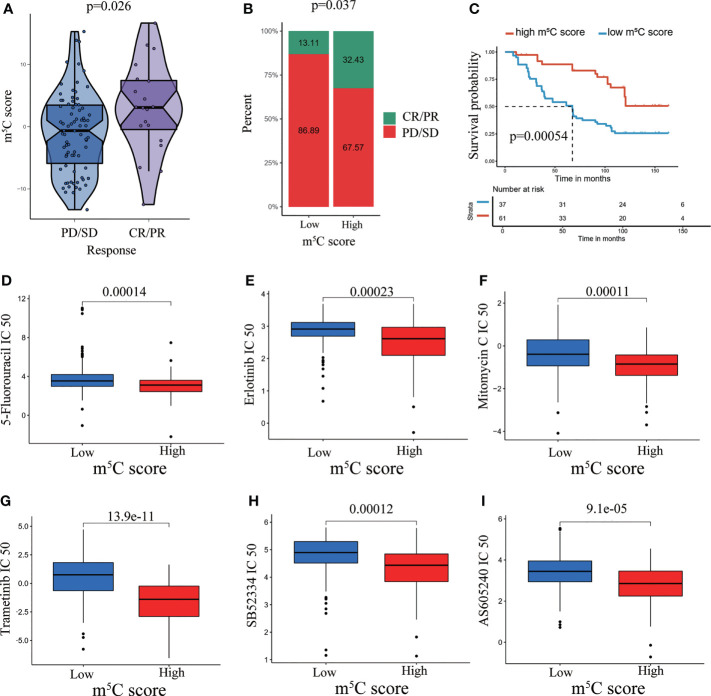
Performance of the m5C score in predicting immunotherapy response and chemotherapy response. **(A)** Violin plots depicting the differences in the m5C score between the CR/PR and SD/PD groups in the GSE91061 cohort. **(B)** Rate of clinical response (complete response [CR]/partial response [PR] and stable disease [SD]/progressive disease [PD]) to anti-PD-1 immunotherapy in the high and low m^5^C score subgroups in the GSE91061 dataset. **(C)** Kaplan–Meier curves for patients with high and low m5C score in the GSE91061 dataset. **(D–I)** Prediction of chemotherapy agent response in high and low m5C score groups.

The m^5^C score reflects regulation and modulation of the immune microenvironment. However, whether the m^5^C score could predict immunotherapy response had not been explored. We examined the utility of the m^5^C score in predicting immunotherapy response. The results showed that patients with high m^5^C score had better sensitivity and response to immunotherapy. A high m^5^C score was related to a higher PR (partial response)/CR (complete response) rate and better OS when compared with low m^5^C score (**Figures 7A–C**). It has been validated chemotherapy agents activated immune responses in patients with tumors, which assisted to improve immunotherapy response (49, 50). It propelled us to examine the chemotherapy agents which were responded differently in high and low m^5^C score group. The results indicated IC50 values of 5-Fluorouracil, Erlotinib, Mitomycin C, Trametinib, SB52334 and AS605240 were decreased in high m5C score group (**Figures 7D–I**). It has been proved some of these regents exerted an inhibitory role on PAAD development (51–53). Combination with these agents may greatly enhance the immunotherapy responsiveness. Our present results confirmed that the m^5^C score is a precise indicator for the prediction of immunotherapy benefit.

### Validation of the Utility of the m^5^C Score in Predicting PAAD Patient Prognosis

**Figure 8:**
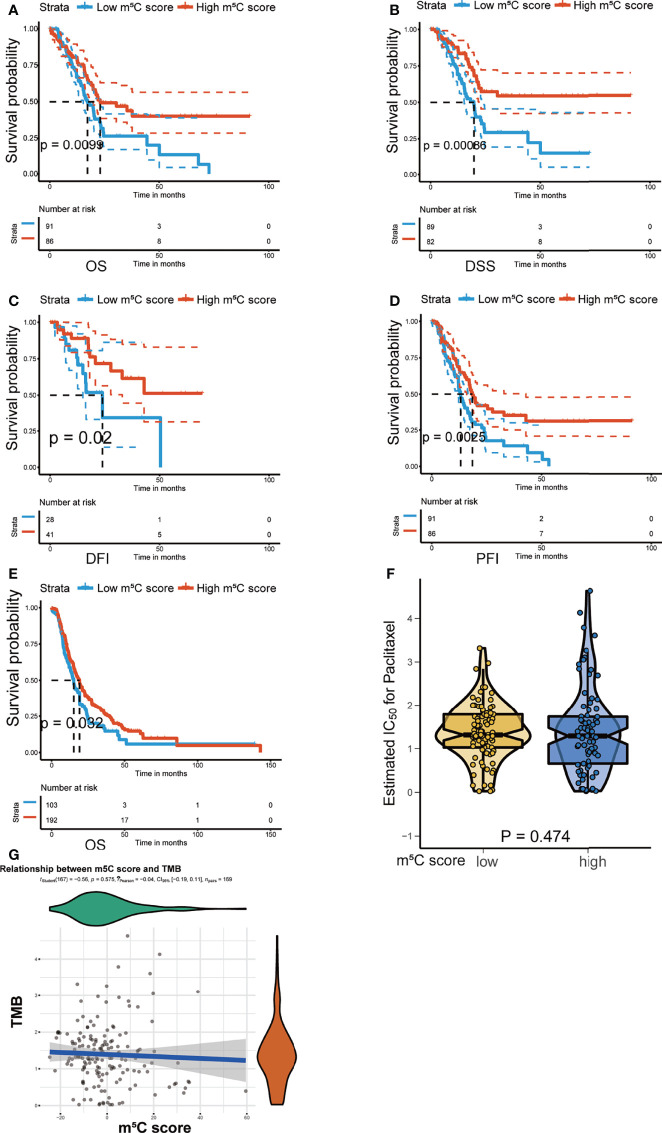
Validation of the utility of the m^5^C score in predicting PAAD patient prognosis. **(A–D)** Kaplan–Meier curves of overall survival (OS), disease-specific survival (DSS), disease-free interval (DFI), and progression-free interval (PFI) for patients with PAAD in the high and low m^5^C score groups in the TCGA PAAD dataset. **(E)** Kaplan–Meier curves of overall survival (OS) for patients with PAAD in the high and low m^5^C score groups in the ICGC-AU and ICGC-CA datasets. **(F, G)** Correlation between the m^5^C score and tumor mutation burden (TMB).

The m^5^C score was verified to predict PAAD patient prognosis in the training cohort. Next, we validated the utility of the m^5^C score using the TCGA PAAD dataset, the ICGC-AU and the ICGC-CA datasets. Consistently, high score was related to better OS, disease-specific survival (DSS), disease-free interval (DFI), and progression-free interval (PFI) in the TCGA PAAD dataset (**Figures 8A–D**). OS was also demonstrated to be related to the m^5^C score in the ICGC-AU and ICGC-CA datasets (**Figure 8E**). We further examined the relationship between the m^5^C score and tumor mutation burden (TMB). TMB has been reported to be related to cancer development and act as an indicator of the immunotherapy response and prognosis (54). In the present study, there was no significant relationship between these two factors, indicating that the m^5^C score was an independent immunotherapy response indicator (**Figures 8F, G**).

### Exploration of the Mechanism Underlying the Utility of the m^5^C Score Model

**Figure 9:**
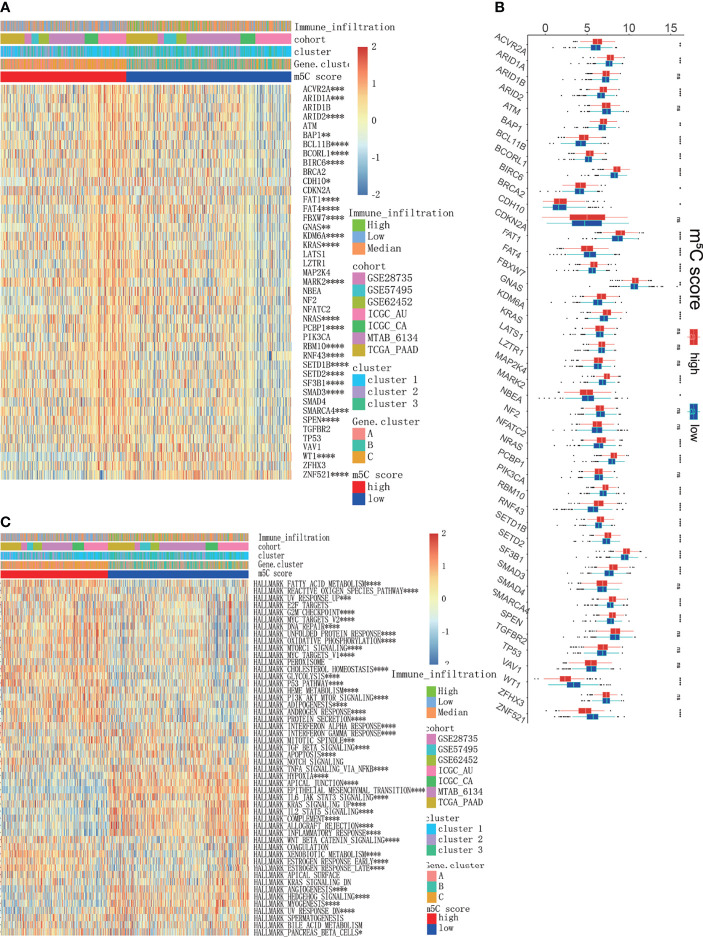
Exploration of the mechanisms underlying the m^5^C score model. **(A, B)** Analysis of cancer driver genes in the high and low m^5^C score groups. **(C)** GSVA results of all PAAD cohorts between the high and low m^5^C score groups, which were also characterized by immune infiltration subtypes, clusters and gene clusters. * represents p value < 0.05, ** represents p value < 0.01, *** represents p value < 0.001, **** represents p value < 0.0001, ns means "not statistically significant".

**Figure 10:**
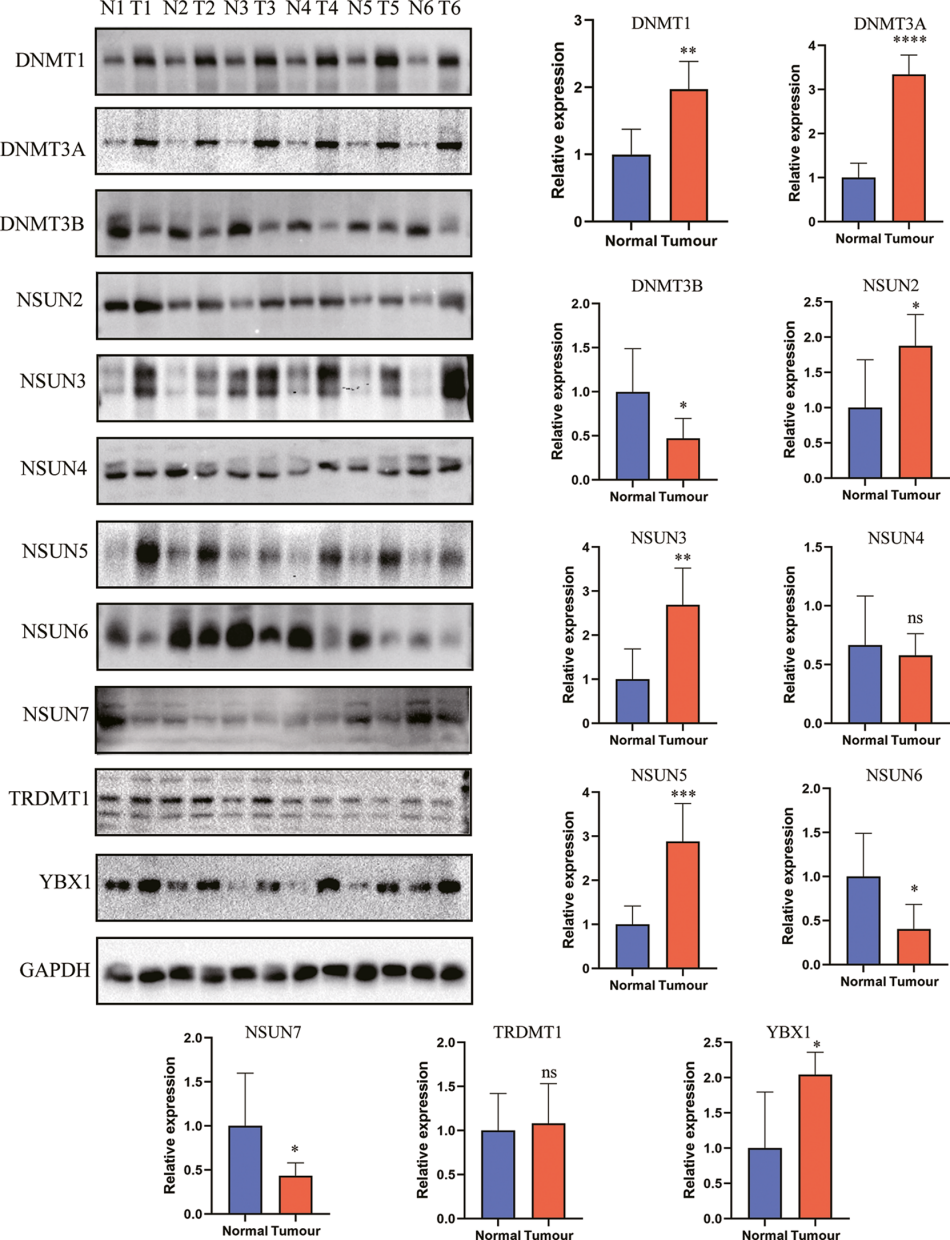
The expression level of m^5^C regulators in PAAD and control tissues. * represents p value < 0.05, ** represents p value < 0.01, *** represents p value < 0.001, **** represents p value < 0.0001, ns means "not statistically significant".

To further uncover the underlying molecular mechanism of the m^5^C score, we analyzed the correlation of cancer-related driver genes with immune infiltration, the m^5^C clusters, the m^5^C gene clusters and the m^5^C score (**Figure 9A**). The results indicated that ACVR2A, ARID1A, ARID2, BAP1, BCL11B, BCORL1, BIRC6, CDH10, FAT1, FAT4, FBXW7, GNAS, KDM6A, KRAS, MARK2, NRAS, PCBP1, RBM10, RNF43, SETD1B, SETD2, SF3B1, SMAD3, SMARCA4, SPEN, WT1 and ZNF521 were differentially expressed between the high and low m^5^C score groups (**Figure 9B**). Gene set variation analysis (GSVA) was also conducted in the high and low m^5^C score groups. The terms reactive oxygen species pathway, DNA repair, unfolded protein response, p53 pathway and PI3K-AKT-MTOR pathway were differentially activated. Moreover, terms related to metabolic processes such as fatty acid metabolism, cholesterol homeostasis, glycolysis, and adipogenesis were significantly altered between the high and low m^5^C score groups. Additionally, inflammation-related pathways were also altered between the high and low m^5^C score groups, including the interferon alpha response, interferon gamma response, TNFα signaling *via* NFκB, IL6-JAK-STAT3 signaling and IL2-STAT5 signaling pathways (**Figure 9C**). Finally, we verified the expression pattern of m^5^C regulators in PAAD patient tissues and corresponding normal tissues (**Figure 10**). Consistently, DNMT1, DNMT3A, NSUN2, NSUN3, NSUN5 and YBX1were increased while DNMT3B, NSUN6 and NSUN7 were decreased in PAAD samples when compared with mRNA and immunohistochemical results. Consistent with previous studies, DNMT1, DNMT3A and YBX1 were increased while NSUN6 was decreased in PAAD tissues when compared with adjacent normal tissues (20, 55–57).

## Discussion

Accumulating evidence suggests that m^5^C regulators play a crucial role in maintaining the physiological activities of cells. Alteration of the expression or distribution of m^5^C regulators results in the pathogenesis and development of cancer (58). m^5^C regulators have been reported to regulate cell proliferation, migration, and chemotherapy resistance and, thus, tumor progression (59). Additionally, m^5^C regulators have been reported to regulate the immune microenvironment. For example, in CD4^+^ T cells from patients with SLE, the levels of the m^5^C writer NSUN2 and methylated m^5^C genes were found to be decreased (13). In cutaneous melanoma, lung adenocarcinoma, lung squamous cell carcinoma, and other cancer types, bioinformatic analysis have shown that m^5^C regulators modulate the tumor immune microenvironment, act as immunotherapy indicators and were related to patients’ prognosis (14–19, 22, 60–64). These studies firmly proved that m^5^C regulators were differentially expressed and modulated the immunotherapy response in multiple cancers. In PAAD, the m^5^C writer NSUN6 has been shown to repress cell proliferation and tumor development (20). However, the effects of m^5^C regulators on the immune microenvironment in PAAD are largely unknown. Comprehensive analysis of m^5^C regulators in PAAD is urgently needed. Understanding the functions and underlying mechanism of m^5^C regulators will help to establish prognostic indicators and provide potential therapeutic strategies in PAAD.

In the present study, we analyzed the expression and genetic alteration patterns of m^5^C regulators in PAAD dataset. The patients were divided into m^5^C clusters and m^5^C gene clusters based on m^5^C regulators expression and m^5^C DEGs, respectively. The results confirmed that m^5^C regulators are related to the immune microenvironment and PAAD patient prognosis. Additionally, we identified a quantitative indicator, the m^5^C score, from the m^5^C gene signatures. m^5^C score was found to predict immunotherapy response and patient prognosis. The underlying driver genes and pathways of m^5^C regulators were further studied.

m^5^C regulators have various expression patterns in different cancers. In hepatocellular carcinoma, NSUN2 expression is increased, and H19 lncRNA stability is maintained to promote cancer development (65). In the present study, we examined the expression patterns of m^5^C regulators in PAAD. The levels of DNMT1, DNMT3A, NSUN2, NSUN3, NSUN5 and YBX1 were increased while those of DNMT3B, NSUN6 and NSUN7 were decreased in PAAD tissues compared to normal tissues. Specifically, DNMT1, DNMT3A and YBX1 were increased while NSUN6 was decreased in PAAD tissues, which are consistent with previous studies in PAAD (20, 55–57). Though genetic alteration rate is low in some cancer types, it plays a critical role to regulate tumor development and predict prognosis (66). In the present study, we founded 51 of the 259 (19.69%) PAAD patients in 2 studies experienced genetic alterations of m^5^C regulators. Despite the low alteration rate, these genetic alterations are potential targets to develop targeted drugs. These results hinted that m^5^C regulators affected PAAD development. However, the underlying mechanism is largely unknown.

The immune microenvironment has been reported to indicate immunotherapy response and patient prognosis (67). In the present study, we analyzed the relationship between m^5^C regulators and the immune microenvironment. Based on the m^5^C regulator expression pattern and m^5^C DEGs, patients were divided into different m^5^C clusters and m^5^C gene clusters. The results showed that m^5^C clusters and m^5^C gene clusters were related to patient OS status, indicating that m^5^C regulators play a significant role in PAAD development. In addition, differences in immune cell infiltration and immune-relevant gene expression between the m^5^C clusters and m^5^C gene clusters were analyzed. Consistently, we found that NK CD56 bright cells were increased and Treg cells were decreased in m^5^C cluster 2 and m^5^C gene cluster C, patients in which had the best prognosis when compared with other groups. NK CD56 bright cells were mainly responsible for the innate immune response. As reported, innate immune response plays a central role in improving immunotherapy response and restraining cancer development (44). However, it can’t be ignored kinds of effector immune cells including CD8^+^ T cells, cytotoxic cells, TCM and TEM were decreased in gene cluster C. It has been reported these immune cells play a vital role in killing tumor cells (68). In the present study, Treg cells were greatly decreased in gene cluster C, which has been proved to suppress the activation and function of effector immune cells in previous study (5). In our opinion, the effector immune cells and immune-suppressive cells antagonize each other and jointly decide the ultimate immunotherapy response of the patients. We speculated that though infiltrating effector immune cells were decreased in gene cluster C, these effector immune cells were activated and functional due to the decreased Treg. Therefore, gene cluster C had the best prognosis. On the contrary, though effector immune cells were increased in other groups, they were dysfunctional for the increased Treg cells. These effector immune cells lost the ability to inhibit tumor growth, thus leading to a worse prognosis. However, this hypothesis remains to be verified in our future study. In conclusion, m^5^C regulators modulate immunotherapy and PAAD progression partly by modulating innate immune response and Treg cells.

RNA modification regulators affect cellular homeostasis by controlling target RNA stability and function. m^5^C regulators have been reported to predict immunotherapy response in multiple cancers (14, 22). However, the predictive and prognostic roles of m^5^C clusters in PAAD are largely unknown. In the present study, we developed a quantitative indicator of m^5^C regulators from the m^5^C gene signatures, called the m^5^C score. We found that the m^5^C score was related to immune cell infiltration and immune-related gene expression. Additionally, the m^5^C score was significantly correlated with immunotherapy response and prognosis indicators, including CAF level, T cell dysfunction, MSI, the TIDE score. These results suggest that the m^5^C score can predict immunotherapy response. We further examine the chemotherapy agents which were responded differentially in high and low m^5^C score group. Chemotherapy agents have been reported to enhance immunotherapy response. It’s promising that combination with these agents is potential strategy to improve immunotherapy responsiveness in PAAD.

TMB has been reported to be related to cancer development and potentially to determine the response to immunotherapy (69). The results of the present study showed that there was no significant correlation between the m^5^C score and TMB, indicating that the m^5^C score is independent of TMB. It has been reported in previous studies low m^5^C score was related to a better prognosis of oral squamous cell carcinoma and papillary thyroid carcinoma patients (19, 70). In our study, high m^5^C score indicated better immunotherapy response and prognosis. It seems inconsistent with previous studies. It’s known PAAD had the unique “cold” immune microenvironment, which is different from that in other cancers. The m^5^C score represented different immune characteristics in different cancer types. Moreover, the same gene always had opposite effects in different types of cancer. It’s another potential cause for the difference. Moreover, gene cluster C had a higher m^5^C score when compared with other gene clusters, which further support our conclusion. In summary, our study provided a reliable and quantitative indicator for predicting PAAD immunotherapy response and prognosis.

Finally, we determined the underlying cancer driver genes and molecular pathways. Our study revealed several cancer driver genes that differed between the high and low m^5^C score groups, including KRAS, FBXW7, and SMAD3. KRAS is the most common cancer driver gene in PAAD (71). The E3 ligase FBXW7 has also been reported to regulate PAAD cell epithelial-mesenchymal transition, ferroptosis and apoptosis (72, 73). GSVA indicated that inflammation and metabolism-relevant pathways were differentially activated between the high and low m^5^C score groups. Inflammation- and metabolism-related pathways have been reported to regulate the immune microenvironment of PAAD (74, 75). The present study implies that m^5^C regulators can modulate the TME by influencing inflammation or metabolism. The present study provides novel potential mechanisms to account for the ability of m^5^C regulators to regulate the TME and predict immunotherapy response. However, the direct target RNAs of m^5^C regulators that modulate inflammation and metabolic processes in PAAD remain to be explored.

In conclusion, we confirmed that m^5^C regulators are related to the immune microenvironment and the prognosis of PAAD patients with an integrated dataset. Additionally, we developed the m^5^C score, a quantitative indicator that can predict immunotherapy response and prognosis. The underlying molecular pathways were examined to further understand the m^5^C score. The results of our study will assist in identifying PAAD patients suitable for tailored immunotherapy strategies.

## Data Availability Statement

The datasets presented in this study can be found in online repositories. The names of the repository/repositories and accession number(s) can be found in the article/**Supplementary Material**.

## Ethics Statement

The studies involving human participants were reviewed and approved by The Research Ethics Committee of Tangdu Hospital. The patients/participants provided their written informed consent to participate in this study.

## Author Contributions

HS and JL conceived and designed the whole project. RW, YG, and PM drafted the manuscript. YS, JM, CZ, CY, JS, and DG collected the data. LZ, SL, and TZ analyzed the data and wrote the manuscript. All authors reviewed and approved the final manuscript.

## Funding

This study was supported by grants from the National Natural Science Foundation of China to HS (No. 81372255) and to RW (No. 81902523).

## Conflict of Interest

The authors declare that the research was conducted in the absence of any commercial or financial relationships that could be construed as a potential conflict of interest.

## Publisher’s Note

All claims expressed in this article are solely those of the authors and do not necessarily represent those of their affiliated organizations, or those of the publisher, the editors and the reviewers. Any product that may be evaluated in this article, or claim that may be made by its manufacturer, is not guaranteed or endorsed by the publisher.

## Supplementary Material

Supplementary Figure 1 The protein expression of m^5^C regulators in PAAD and normal tissues obtained from the Human Protein Atlas (HPA) database.

Supplementary Figure 2 m^5^C cluster construction. **(A-H)** Consensus clustering matrix of all 930 PAAD patients for each k (k=2 to 9). **(I)** Consensus clustering cumulative distribution function (CDF) for k=2 to 9. **(J)** Relative change in the area under the CDF curve for k=2 to 9;.**(K)** Tracking plot for k=2 to 9.

Supplementary Figure 3 Construction of m^5^C gene clusters. **(A-I)** Consensus clustering matrix of all 930 PAAD patients for each k (k=2 to 9). **(J)** Consensus clustering cumulative distribution function (CDF) for k=2 to 9. **(K)** Relative change in the area under the CDF curve for k=2 to 9. **(L)** Tracking plot for k=2 to 9.

Supplementary Figure 4 A heatmap was constructed to delineate the correlation between the m^5^C score and the TME.
