# Supplementary figures and images for: Comprehensive Analysis of 5-Methylcytosine (m5C) Regulators and the Immune Microenvironment in Pancreatic Adenocarcinoma to Aid Immunotherapy
Source: Front Oncol. 2022 Mar 31;12:851766. doi: 10.3389/fonc.2022.851766 (PMC9009261; doi:10.3389/fonc.2022.851766)

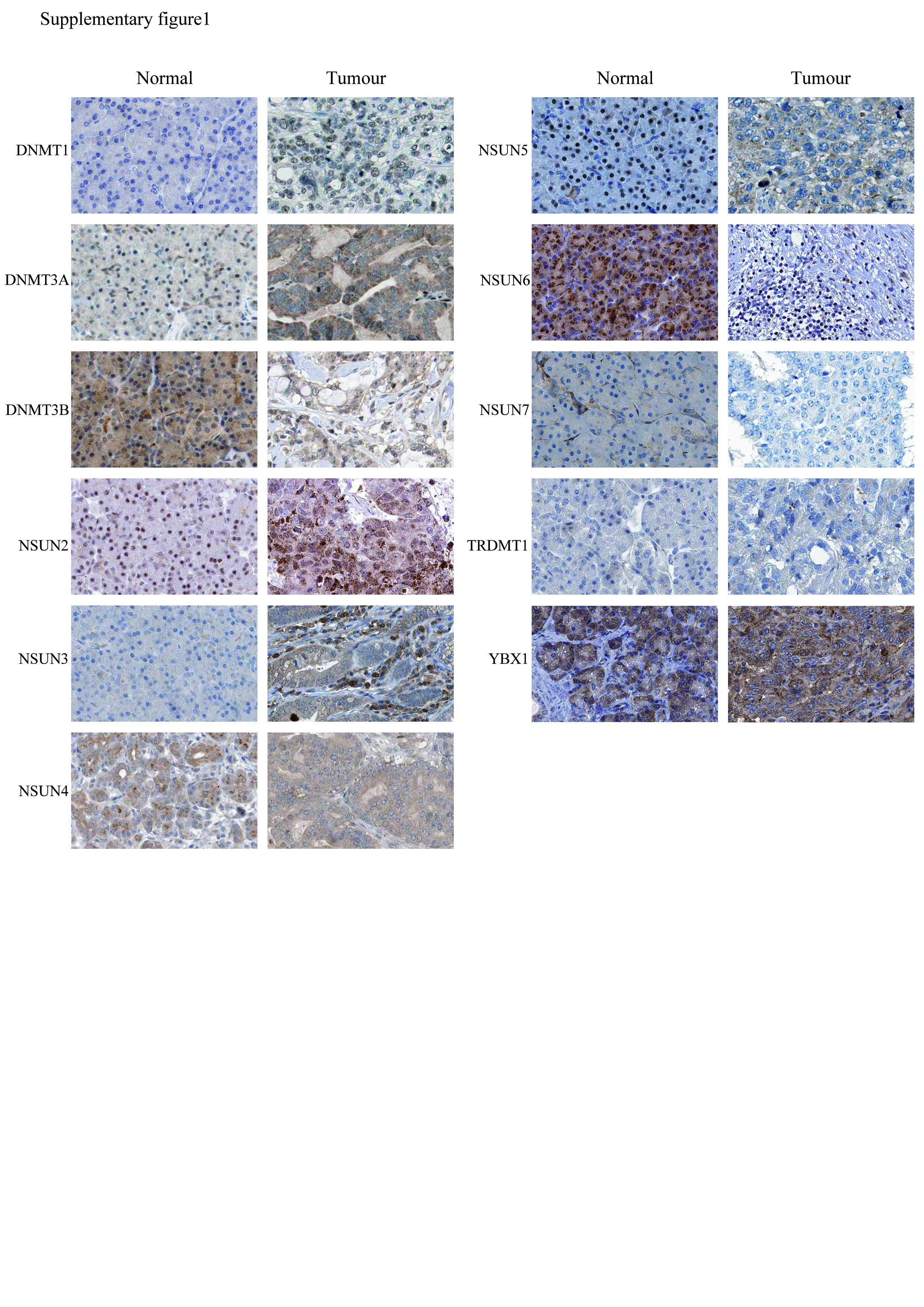

Supplement: Supplementary Figure 1 — The protein expression of m5C regulators in PAAD and normal tissues obtained from the Human Protein Atlas (HPA) database. [file Image_3.tif]

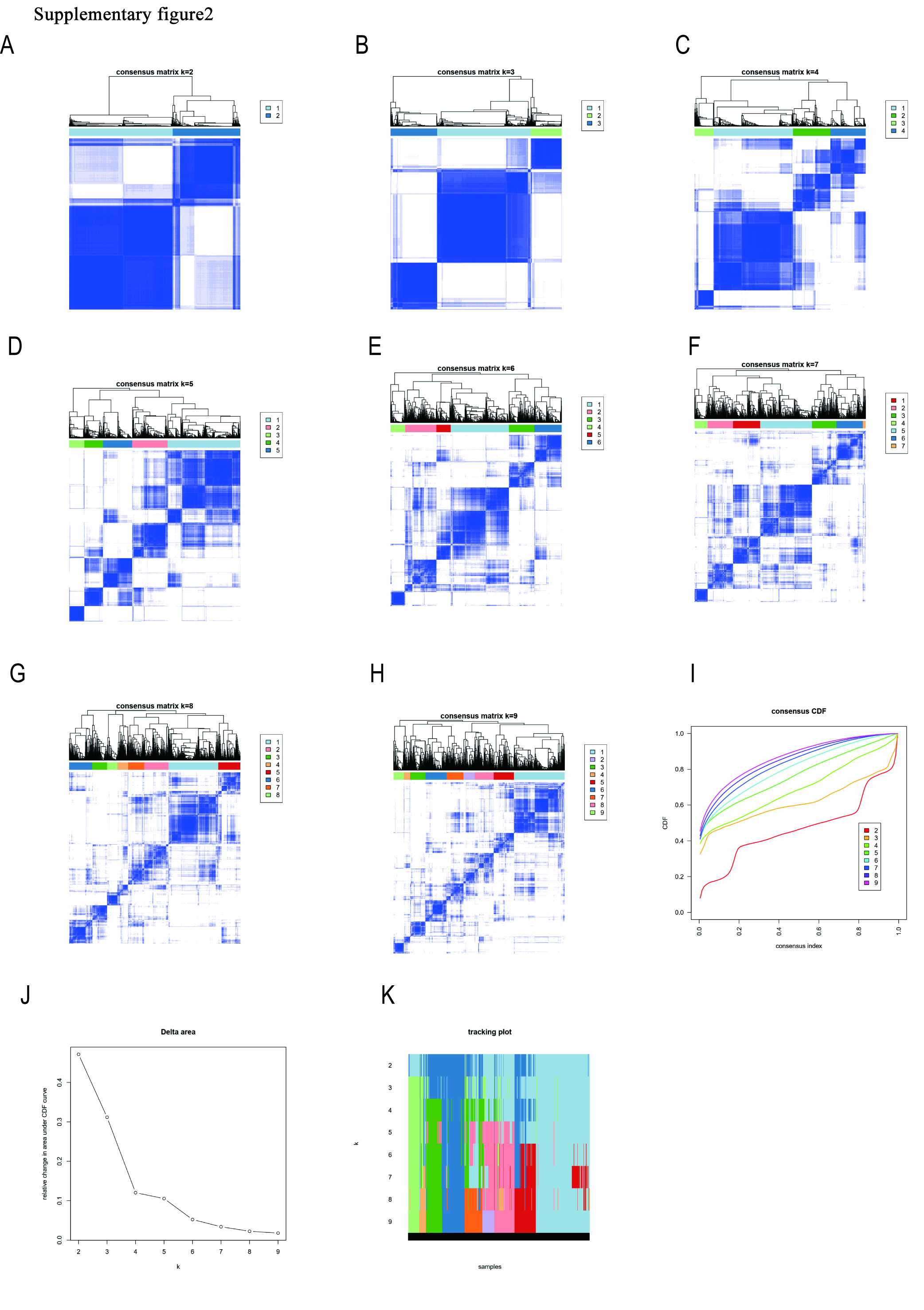

Supplement: Supplementary Figure 2 — m5C cluster construction. (A-H) Consensus clustering matrix of all 930 PAAD patients for each k (k=2 to 9). (I) Consensus clustering cumulative distribution function (CDF) for k=2 to 9. (J) Relative change in the area under the CDF curve for k=2 to 9;.(K) Tracking plot for k=2 to 9. [file Image_4.tif]

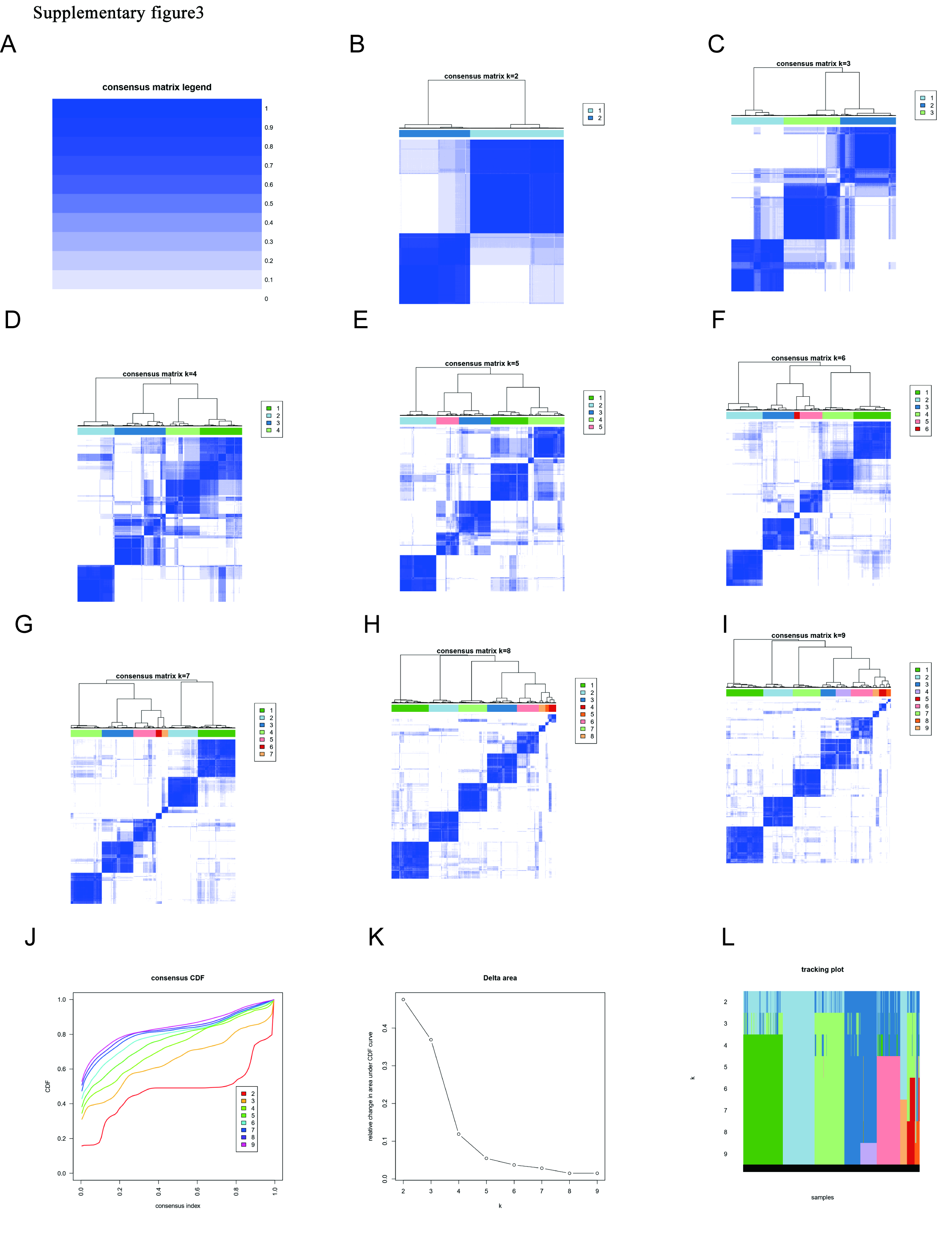

Supplement: Supplementary Figure 3 — Construction of m5C gene clusters. (A-I) Consensus clustering matrix of all 930 PAAD patients for each k (k=2 to 9). (J) Consensus clustering cumulative distribution function (CDF) for k=2 to 9. (K) Relative change in the area under the CDF curve for k=2 to 9. (L) Tracking plot for k=2 to 9. [file Image_5.tif]

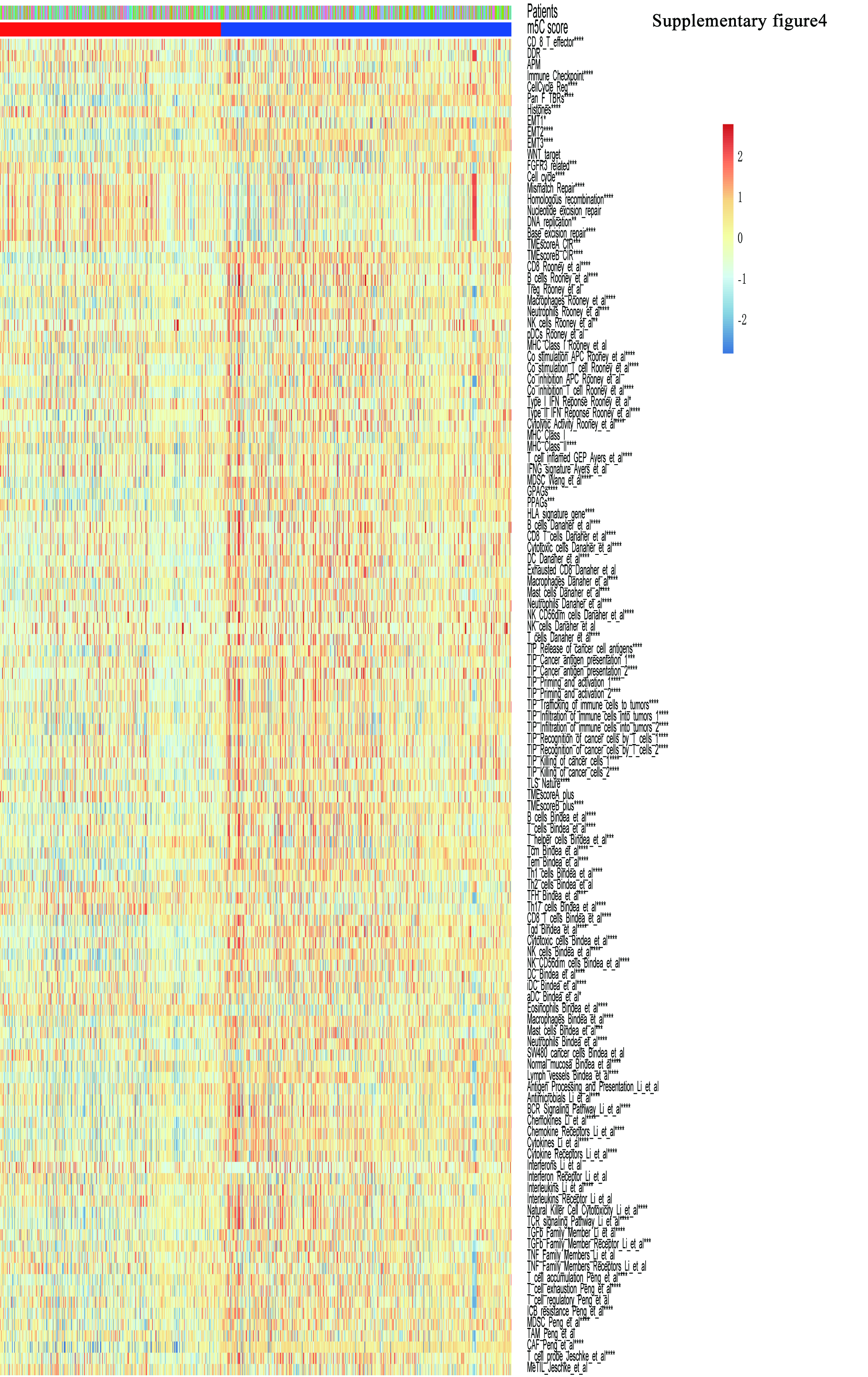

Supplement: Supplementary Figure 4 — A heatmap was constructed to delineate the correlation between the m5C score and the TME. [file Image_6.tif]
